# The global network antenatal corticosteroids trial: impact on stillbirth

**DOI:** 10.1186/s12978-016-0174-4

**Published:** 2016-06-02

**Authors:** Robert L. Goldenberg, Vanessa R. Thorsten, Fernando Althabe, Sarah Saleem, Ana Garces, Waldemar A. Carlo, Omrana Pasha, Elwyn Chomba, Shivaprasad Goudar, Fabian Esamai, Nancy F. Krebs, Richard J. Derman, Edward A. Liechty, Archana Patel, Patricia L. Hibberd, Pierre M. Buekens, Marion Koso-Thomas, Menachem Miodovnik, Alan H. Jobe, Dennis D. Wallace, José M. Belizán, Elizabeth M. McClure

**Affiliations:** Columbia University, New York, NY USA; RTI International, Durham, NC USA; IECS, Buenos Aires, Argentina; Aga Khan University, Karachi, Pakistan; Guatemala, Guatemala; University of Alabama at Birmingham, Birmingham, AL USA; University Teaching Hospital, Lusaka, Zambia; KLE University’s JN Medical College,, Belgaum, India; Moi University, Eldoret, Kenya; University of Colorado School of Medicine, Denver, CO USA; Christiana Care, Newark, DE USA; Indiana University, Indianapolis, IN USA; Lata Medical Research Foundation, Nagpur, India; Massachusetts General Hospital, Boston, MA USA; Tulane School of Public Health and Tropical Medicine, New Orleans, LA USA; Eunice Kennedy Shriver National Institute of Child Health and Human Development, Bethesda, MD USA; Cincinnati Children’s Hospital, Cincinnati, OH USA; Department of Obstetrics and Gynecology, Columbia University, New York, NY USA

## Abstract

**Background:**

Antenatal corticosteroids are commonly used to reduce neonatal mortality, but most research to date has been in high-resource settings and few studies have evaluated its impact on stillbirth. In the Antenatal Corticosteroids Trial (ACT), a multi-country trial to assess impact of a multi-faceted intervention including antenatal corticosteroids to reduce neonatal mortality associated with preterm birth, we found an overall increase in 28-day neonatal mortality and stillbirth associated with the intervention.

**Methods:**

The ACT was a cluster-randomized trial conducted in 102 clusters across 7 research sites in 6 countries (India [2 sites], Pakistan, Zambia, Kenya, Guatemala and Argentina), comparing an intervention to train birth attendants at all levels of the health system to identify women at risk of preterm birth, administer corticosteroids and refer women at risk. Because of inadequate gestational age dating, the <5^th^ percentile birth weight was used as a proxy for preterm birth. A pre-specified secondary outcome of the trial was stillbirth.

**Results:**

After adjusting for the pre-trial imbalance in stillbirth rates, the ACT intervention was associated with a non-significant increased risk of stillbirth (aRR 1.08, 95 % CI, 0.99–1.17, *p*–0.073). Additionally, the stillbirth rate was higher in the term births (1.20 95 % CI 1.06–1.37, 0.004) and among those with signs of maceration (RR 1.18 (1.04–1.35), *p* = 0.013) in the intervention vs. control clusters. Differences in obstetric care favored the control clusters and maternal infection was likely more common in the intervention clusters.

**Conclusions:**

In this pragmatic trial, limited data were available to identify the causes of the increase in stillbirths in the intervention clusters. A higher rate of stillbirth in the intervention clusters prior to the trial, differences in obstetric care and an increase in maternal infection are potential explanations for the observed increase in stillbirths in the intervention clusters during the trial.

**Trial registration:**

clinicaltrials.gov (NCT01084096)

## Background

The Antenatal Corticosteroid Trial (ACT) was a cluster randomized trial performed in 7 sites in 6 low and middle income countries (LMIC) [1, 2]. The primary outcomes for that trial were to determine whether a multicomponent intervention increased the administration of antenatal corticosteroids (ACS) in the women at risk of preterm birth and then reduced neonatal mortality among that group. The primary outcome of neonatal mortality outcome was assessed among infants born at less than the site specific 5^th^ percentile birth weight and was used as a surrogate measure for preterm birth. The intervention increased ACS use in the treatment clusters (46 % vs.10 % in <5^th^ percentile births), but being in the intervention clusters was not associated with an improvement in neonatal mortality in the < 5^th^ percentile infants [1]. An unexpected finding was that neonatal mortality was higher overall in the intervention clusters, with the excess mortality occurring in infants born at ≥25^th^ percentile site-specific birth weight.

A pre-specified secondary outcome was the stillbirth rate, which was higher in the intervention clusters compared to the control clusters [2]. In this paper, we explore potential mechanisms which might have resulted in the higher stillbirth rate in the intervention clusters. Of note, ACT was a “pragmatic” trial and not designed to evaluate mechanisms leading to specific pregnancy outcomes, especially those such as stillbirth that were not a primary endpoint. We also emphasize that compared to prior trials demonstrating efficacy of ACS which were all individually randomized and conducted in in high and some middle income country hospitals with good obstetric and neonatal care, this cluster randomized trial enrolled more than 100,000 subjects in countries where half the women delivered at home or in low-level community clinics [3, 4].

The hospitals in these areas rarely provided comprehensive obstetric or intensive newborn services and rarely monitored the fetuses during labor [5]. During the time that ACT was initiated, ultrasound gestational age dating was generally unavailable for women in these sites. For that reason, the target group for the primary neonatal outcome was not determined by gestational age, but by a birth weight < 5^th^ percentile for babies born at that site. Because placental examinations and autopsies were not available, little data are available to assess the causes of the stillbirths. Nevertheless, because of the unexpected excess of stillbirths in the intervention compared to the control clusters during the ACT trial, we used available data to explore potential reasons for the higher rate of stillbirths in the intervention clusters.

## Methods

This is a secondary analysis of data collected during the ACT study focusing on stillbirths. A stillbirth was defined as a baby born at ≥500 g or an estimated 20 weeks gestational age without a heartbeat, respirations or signs of movement [6]. ACT was a cluster-randomized trial undertaken in specified geographic clusters each with approximately 500 births per year in sites in Argentina, Guatemala, Kenya, Zambia, Pakistan, and in Belgaum and Nagpur, India. The study intervention and methods are described in detail elsewhere [1]. In brief, the study clusters were defined by each site and then randomized by the data center, RTI International (RTI). To randomize clusters, RTI created strata based on the study site and neonatal mortality rates using an ongoing registry; however the stillbirth rates in the individual clusters were not compared prior to the trial nor considered in the randomization process. The ACT outcome data were collected independently of the intervention team by trained registry administrators (RAs) in a prospective maternal and newborn health (MNH) registry, which registered pregnant women residing within the study clusters and recorded their outcomes. These included all deliveries, regardless of location in home, clinic or hospital. In addition, in the ACT intervention clusters, trained staff at each site collected process data on the use of ACS. The study was overseen by a research team at each of the study sites and a central study steering committee.

All health providers in intervention clusters were trained to identify women presenting before 36 weeks’ gestation with signs of labor, preterm premature rupture of membranes, pre-eclampsia or eclampsia, and obstetric hemorrhage as at high risk of preterm birth and potential candidates for ACS. Providers were trained to assess gestational age by use of an algorithm that included last menstrual period (LMP) and estimated delivery date, or uterine height if neither the LMP nor estimated delivery date were known. Uterine height was measured using a color-coded tape that was developed and validated to measure uterine height, with a red zone indicating an estimated gestational age younger than 36 weeks and 0 days [7]. Ultrasound was considered to be the best estimate for gestational age determination, but was rarely available. Posters were displayed at facilities in the intervention group and discs were designed and distributed to calculate gestational age on the basis of LMP or estimated delivery date. The posters, discs and tapes also served as reminders about signs of risk. This gestational age assessment was used to determine if a woman was at high risk for preterm birth. For women determined to be eligible, providers were trained to provide one course of ACS (dexamethasone).

For intervention and control clusters, stillbirths were classified as <5^th^ percentile based on measured birth weight and estimated weights by clinical assessment when measured weights were missing being less than the site-specific cut off. Those without any measure of birth weight were coded as <5^th^ percentile. Additionally, site-specific birth weight percentile bands and broad birth weight groups were determined from measured and estimated birth weights collected by the RAs. If a stillbirth did not have a measured weight, estimated weights were used when they fit in one of the percentile bands or otherwise was recorded as missing. Preterm delivery (<37 weeks vs ≥ 37 weeks) was determined by an algorithm which used the estimated delivery date and last menstrual period recorded by the RAs, and site-specific 95^th^ percentile for birth weight at 36 weeks.

Stillbirths in low-income countries are often classified by maceration or fresh status [6]. The RAs were trained to determine maceration status using pictures of macerated stillbirths (e.g., peeling skin) in training sessions. Fresh stillbirths are generally considered to have occurred within 12 h of delivery and generally during the intrapartum period. Most of these deaths are believed to be due to asphyxia occurring during labor. Macerated stillbirths are generally considered to have died before labor [8]. The deaths in macerated fetuses are considered due to a variety of causes including infection [9].

In the analysis phase, suspected maternal infection was assessed by using a composite of process outcomes including maternal receipt of antibiotics plus hospital admission or referral, and receipt of intravenous fluids, surgery, or other treatment related to infection [2]. Additionally, women with postpartum signs and symptoms of severe sepsis with admission to hospital or sepsis as the primary cause of maternal death were included. The definition also included evidence of antepartum or post-partum infection for mothers with infants with a birth weight less than 2500 g. This algorithm was not used clinically.

The trial population included all births, whether stillborn or live born. The trial period included births between October 2011 and March 2014, depending on each site’s 18-month enrollment period, with most births occurring in 2012 and 2013. Because the MNH registry was initiated prior to the trial, stillbirth rates and process data were available in each cluster for at least a year prior to the initiation of the study [10]. The pretrial period occurred in 2010 and 2011 (four clusters in Belgaum, India were added in 2011).

### Statistical analyses

Generalized linear models were used to evaluate the relationship between covariates and the outcomes of interest and to develop point and interval estimates of relative risk (RR) associated with these risk factors. Generalized estimating equations were used to account for the correlation of outcomes within cluster to develop appropriate confidence intervals. Models were log binomial when possible, otherwise Poisson models were utilized. Models were adjusted for randomization strata. All analyses were done by RTI International with SAS versions 9.3 and 9.4 (SAS Institute, Cary, NC, USA).

### Ethical approval

The ACT trial was reviewed and approved by the ethics committees at each site, the World Health Organization and the NICHD. All women provided informed consent prior to participation. Trial registration was at clinicaltrials.gov (NCT01084096).

## Results

We previously reported the stillbirth rates in the intervention and control arms of the study [2]. Overall, there were a total of 48,219 women and 48,698 babies in the ACT intervention clusters and 51,523 women and 52,007 babies in the control clusters. There were a total of 1,304 stillbirths in the treatment clusters (26.8 per 1000 births) vs 1,264 in the control clusters (24.3 per 1000 births) with (RR 1.11, 95 % CI 1.02–1.22, *p* = 0.0181) (Table 1) Using similar registry data for the period prior to the trial (2010), the stillbirth rate was 31.8 per 1000 births in the intervention clusters and 27.6 per 1000 births in the control clusters (RR 1.17, 95 % CI 1.06,1.29, *p* = 0.0026). When we adjusted for the pretrial stillbirth rate and randomization strata, the risk of stillbirth during the trial period associated with being in the intervention clusters decreased from 1.11 to 1.08 (95 % CI, 0.99–1.17, *p* =0.073). Thus, the results were in the same direction but no longer statistically significant.

**Table 1:** Stillbirth rates during the pretrial (2010) and trial periods by ACT intervention group and site

Pretrial period																		
	Argentina		Guatemala		Pakistan		Belgaum		Nagpur		Kenya		Zambia		Total			
Characteristic^a^	Int	Cntl	Int	Cntl	Int	Cntl	Int	Cntl	Int	Cntl	Int	Cntl	Int	Cntl	Int	Cntl	RR (95 %)^b^	*p*-value
All babies, N	1,439	1,437	2,981	2,321	5,481	7,101	8,675	9,649	5,206	4,958	3,994	5,209	2,986	4,188	30,762	34,863		
Stillbirths, N (Rate/1000)^a^	28 (19.5)	22 (15.3)	67 (22.5)	50 (21.5)	295 (53.8)	320 (45.1)	255 (29.4)	252 (26.1)	148 (28.4)	126 (25.4)	84 (21.0)	102 (19.6)	101 (33.8)	90 (21.5)	978 (31.8)	962 (27.6)	1.17 (1.06,1.29)	0.003
<5th percentile for weight babies, N	81	99	124	123	717	746	358	389	267	299	167	217	176	214	1,890	2,087		
<5th percentile stillbirths, N (Rate/1000)^a^	16 (197.5)	12 (121.2)	27 (217.7)	18 (146.3)	214 (298.5)	200 (268.1)	150 (419.0)	154 (395.9)	90 (337.1)	87 (291.0)	36 (215.6)	39 (179.7)	54 (306.8)	59 (275.7)	587 (310.6)	569 (272.6)	1.15 (1.04, 1.28)	0.008
Trial period																		
	Argentina		Guatemala		Pakistan		Belgaum		Nagpur		Kenya		Zambia		Total			
Characteristic^a^	Int	Cntl	Int	Cntl	Int	Cntl	Int	Cntl	Int	Cntl	Int	Cntl	Int	Cntl	Int	Cntl	RR (95 %)^b^	*p*-value
All babies, N	2,122	2,311	5,863	3,978	7,800	8,315	15,022	16,774	7,570	7,655	5,993	7,187	4,328	5,787	48,698	52,007		
Stillbirths, N (Rate/1000)^a^	25 (11.8)	31 (13.4)	113 (19.3)	73 (18.4)	388 (49.7)	351 (42.2)	376 (25.0)	382 (22.8)	158 (20.9)	181 (23.6)	145 (24.2)	154 (21.4)	99 (22.9)	92 (15.9)	1,304 (26.8)	1,264 (24.3)	1.11 (1.02–1.22)	0.018
																	Adjusted for pretrial SB rate 1.08 (0.99,1.17)	0.073
<5th percentile for weight babies, N	105	149	390	192	984	890	777	874	464	377	288	252	260	263	3,268	2,997		
<5th percentile stillbirths, N (Rate/1000)^a^	14 (133.3)	18 (120.8)	44 (112.8)	26 (135.4)	224 (227.6)	203 (228.1)	244 (314.0)	256 (292.9)	107 (230.6)	122 (323.6)	53 (184.0)	63 (250.0)	62 (238.5)	51 (193.9)	748 (228.9)	739 (246.6)	0.99 (0.90–1.09)	0.813
																	Adjusted for pretrial <5th percentile for weight SB rate 0.97 (0.89, 1.05)	0.409

^a^The trial period included births between October 2011 and March 2014, depending on each site’s 18-month enrollment period, with most births occurring in 2012 and 2013. Most births included in the pretrial period occurred in 2010. However, for the four clusters in Belgaum, India which were added after 2010, births from May 2011-May 2012 were included. Stillbirths are ≥ 20 weeks gestational age or ≥ 500 g. The denominator for the stillbirth rate is stillbirths + live births

^b^Generalized linear models were used to evaluate the relationship between covariates and the outcomes of interest and to develop point and interval estimates of relative risk (RR) associated with these risk factors. Generalized estimating equations were used to account for the correlation of outcomes within cluster to develop appropriate confidence intervals. Models were log binomial when possible, otherwise Poisson models were utilized. Models were adjusted for randomization strata

Prior to the trial, the stillbirth rate in the <5^th^ percentile births was 310.6 per 1000 in the intervention group and 272.6 per 1000 births in the control group (RR 1.15, 95 % CI 1.04–1.28, *p* = 0.008) (Table 1). During the trial, the stillbirth rate in the intervention clusters in the <5^th^ percentile births was 228.9/1000 births while in the < 5^th^ percentile births in the control clusters, the stillbirth rate was 246.6/1000 births (RR 0.99, 95 % CI 0.90–1.09, *p* = 0.813). Adjusting for the pretrial stillbirth rate and randomization strata, the stillbirth risk among the <5^th^ percentile births remained similar (RR 0.97, 95 % CI 0.89–1.05, *p* = 0.409).

We evaluated the stillbirth rates between the intervention and control groups by site in the pretrial and trial periods. As shown in Table 1, the stillbirth rates fell substantially in all sites but Kenya from the pretrial to the trial period. The overall reductions in stillbirth rates were by 5.0/1,000 births in the intervention clusters and 3.3/1,000 births in the control clusters. During the trial period, 5 of the 7 sites including Pakistan, Belgaum, Zambia, Kenya and Guatemala had a higher stillbirth rate in the intervention compared to the control clusters, while Nagpur and Argentina had lower stillbirth rates in the intervention clusters. The higher stillbirth rates in the intervention clusters in Pakistan (49.7 vs. 42.2 per 1000, *p* = 0.009) and in Zambia (22.9 vs.15.9 per 1000, *p* = 0.020) were statistically significant as was the lower stillbirth rate in the intervention clusters compared to the control clusters in Argentina (11.8 vs. 13.4 per 1000, *p* = 0.006). In the <5^th^ percentile births, Zambia had a higher stillbirth rate in the intervention clusters that was nearly significant (238.5 vs. 193.9 per 1000, *p* = 0.0561 while Kenya had a lower stillbirth rate in the intervention clusters that also was nearly significant (184.0 vs 250.0 per 1000, *p* = 0.066).

We next explored whether there were differences between stillbirth rates by sex in the intervention compared to the control clusters. There were 667 male stillbirths in the intervention clusters with a rate of 26.6 per 1000 births and 672 stillbirth males in the control clusters for a rate of 25.0 per/1000 births (Table 2). There were 561 female stillbirths in the intervention clusters (23.8 per 1000) and 540 female stillbirths for a rate of 21.6 per 1000 births in the control clusters. Therefore, the stillbirth rates were higher in male infants and the differences in stillbirth rates between male and female fetuses were similar in the intervention and control clusters.

**Table 2:** Stillbirth characteristics by ACT intervention group

			RR (95 % CI)^d^	*p*-value
Characteristic ^a^	Intervention	Control		
Babies, N	48,698	52,007	--	--
Stillbirths (SB), N	1,304	1,264	--	--
Male SB rate, N (Rate/1000)	667 (26.6)	672 (25.0)	1.10 (0.99, 1.22)	0.086^d^
Female SB rate, N (Rate/1000)	561 (23.8)	540 (21.6)	1.08 (0.96, 1.22)	0.177^d^
Birth weight percentile ^b^, N (% of stillbirths)	1,060	1,014		0.211^e^
< 5th	601 (56.7)	612 (60.4)		
5–24	161 (15.2)	139 (13.7)		
25–49	110 (10.4)	102 (10.1)		
50–74	83 (7.8)	74 (7.3)		
75+	105 (9.9)	87 (8.6)		
Birth weight categorized^c^, N	1,259	1,218		
< 1000 g SB rate, N (Rate/1000)	161 (631.4)	197 (693.7)	0.93 (0.83, 1.05)	0.239^d^
1000–1499 g SB rate, N (Rate/1000)	272 (443.0)	215 (378.5)	1.22 (1.10, 1.36)	0.0003^d^
1500–2499 g SB rate, N (Rate/1000)	360 (51.6)	365 (57.4)	0.91 (0.78, 1.06)	0.218^d^
2500+ g SB rate, N (Rate/1000)	466 (11.4)	441 (9.9)	1.18 (1.04, 1.33)	0.008^d^
Preterm SB rate, N (Rate/1000)	645 (116.6)	681 (127.8)	0.93 (0.83, 1.04)	0.182^d^
Term SB rate, N (Rate/1000)	535 (12.8)	477 (10.6)	1.20 (1.06,1.37)	0.004^d^
Macerated, N (% of stillbirths)	467 (35.8)	385 (30.5)	1.18 (1.04, 1.35)	0.013^f^
Women in the treatment group identified by the intervention as high risk who received steroids and delivered a stillborn baby				
Time since 1st dose to delivery, N (%)	271			
Less Than 2 Days	83 (30.6)			
2–7 Days	53 (19.6)			
8–30 Days	61 (22.5)			
More than one month	74 (27.3)			

^a^Stillbirth ≥ 20 weeks GA or ≥ 500 g. The denominator for the stillbirth rate is stillbirths + live births

^b^Site-specific birth weight percentile (measured and estimated weights combined)

^c^Birth weight groups (measured and estimated weights combined)

^d^Generalized linear models were used to evaluate the relationship between ACT intervention group and stillbirth and to develop point and interval estimates of relative risk (RR) associated with these risk factors. Generalized estimating equations were used to account for the correlation of outcomes within cluster to develop appropriate confidence intervals. Models were log binomial when possible, otherwise Poisson models were utilized. Models were adjusted for randomization strata

^e^Mantle Hansel chi-square test of ACT intervention group by birth weight percentile band adjusted for randomization strata

^f^Generalized linear model was used to evaluate the relationship between ACT intervention group and maceration among stillbirths and to develop point and interval estimates of relative risk (RR) associated with these risk factors. Generalized estimating equations were used to account for the correlation of outcomes within cluster to develop appropriate confidence intervals. Model was log binomial and was adjusted for randomization strata

Table 2 also provides the birth weight percentile and birth weight group distributions in the intervention and control clusters among the stillbirths. Site specific birth weight percentile bands could not be categorized for 244 (19 %) of intervention babies and 250 (20 %) of control babies. Stillbirths in the treatment and control groups were similarly distributed in the 25^th^ - 49^th^ and 50^th^ – 74^th^ birth weight percentile bands. Among the intervention group there were more stillbirths in the 5^th^ - 24^th^ percentile band and the 75th or greater percentile band than the control group. However, when adjusted by randomization strata, these differences in the distribution by treatment group were not statistically significant (*p* = 0.2114). There were fewer stillbirths missing from the broad birth weight groups (3 % of the intervention and 4 % of the control stillbirths did not have a measured or estimated birth weight). When compared to the control group, the intervention cluster stillbirth rates were lower, but not significantly so, in the <1000 g (*p* = 0.2385) and 1,500-2,499 g (*p* = 0.2177) groups and significantly higher in the 1,000-1,499 g (*p* = 0.0003) and ≥2,500 g groups (*p* = 0.0078).

Conceding that our gestational age data were not optimal, we nevertheless attempted to evaluate and compare stillbirth rates by whether the fetuses in the intervention and control clusters were preterm or term. When the babies were divided into term and preterm groups, stillbirth rates were lower but not significantly so in births classified as preterm (116.6 vs 127.8 per 1000 births (RR 0.93, 95 % CI 0.83, 1.04, *p* = 0.182) (Table 2) and significantly higher in births classified as term in the treatment vs control clusters, 12.8 vs 10.6 per 1000 births (RR 1 · 20, 95 % CI 1 · 06, 1 · 37, *p* = 0.004).

Considering these three analyses, the most consistent finding was a higher rate of stillbirth in the heavier, and most likely term fetuses in the intervention clusters. The results for the lighter less mature infants were inconsistent, but some of the results suggest that there may have been a small reduction in stillbirths in some of the low birth weight and preterm groups in the intervention clusters.

We compared the maceration status of the stillbirths by treatment group (Table 2). Over one third of stillbirths (35.8 %) in the treatment and 30.5 % in the control group were macerated. When we modeled macerated status among the stillbirths, we found that the intervention cluster stillbirths were significantly more likely to be macerated (RR 1.18, 95 % CI 1.04, 1.35, *p* = 0.013). In every percentile group, birth weight group and gestational age group, maceration was more common in the intervention clusters while fresh stillbirths were more common in the control clusters (Table 3).

**Table 3:** Macerated and non-macerated stillbirths by ACT intervention group

	Total	
Characteristic, N (%)	Intervention	Control
All stillbirths, N	1,304	1,264
Maceration	467 (35.8)	385 (30.5)
No maceration	837 (64.2)	879 (69.5)
Birth weight percentile^a^		
< 5th	601	612
Maceration	251 (41.8)	222 (36.3)
No maceration	350 (58.2)	390 (63.7)
5–24	161	139
Maceration	54 (33.5)	38 (27.3)
No maceration	107 (66.5)	101 (72.7)
25–49	110	102
Maceration	28 (25.5)	22 (21.6)
No maceration	82 (74.5)	80 (78.4)
50–74	83	74
Maceration	21 (25.3)	12 (16.2)
No maceration	62 (74.7)	62 (83.8)
75+	105	87
Maceration	20 (19.0)	11 (12.6)
No maceration	85 (81.0)	76 (87.4)
Birth weight categorized^b^		
< 1000 g	161	197
Maceration	70 (43.5)	71 (36.0)
No maceration	91 (56.5)	126 (64.0)
1000–1499 g	272	215
Maceration	122 (44.9)	82 (38.1)
No maceration	150 (55.1)	133 (61.9)
1500–2499 g	360	365
Maceration	139 (38.6)	127 (34.8)
No maceration	221 (61.4)	238 (65.2)
2500+ g	466	441
Maceration	116 (24.9)	94 (21.3)
No maceration	350 (75.1)	347 (78.7)
Preterm	645	681
Maceration	258 (40.0)	226 (33.2)
No maceration	387 (60.0)	455 (66.8)

^a^Site-specific birth weight percentile (measured and estimated weights combined)

^b^Birth weight (measured and estimated weights combined)

We also explored when the stillbirths occurred in relationship to the timing of the first ACS injection, but had this information only for the intervention clusters. Table 2 shows that half of the mothers of stillbirths who received ACS delivered within the first week after the ACS injection, (31 % at <2 days) with about a quarter of the deliveries each occurring at 8–28 days and > 28 days. Thus, many of the mothers of stillbirths delivered in relatively close proximity to receiving the ACS dose. Conversely, about half delivered a week or more after the ACS administration.

We evaluated various measures of care in the intervention and control clusters, both in the pretrial period and during the trial in an attempt to discover whether differences in care or changes in care during the intervention period might explain part of the difference in stillbirth rates between the intervention and control clusters. Because we had pretrial registry data, we were able to evaluate if differences in care observed during the trial predated the trial. To sum up the data presented in Table 4, there were differences in the prenatal care and obstetric care between the intervention and control clusters. Women in the intervention clusters were more likely to be attended by nurses during delivery than those in the control clusters (38 % vs. 30 %) and less likely to be attended by physicians (39.7 % vs. 45.1 %). More women in the intervention clusters than in the control clusters delivered in clinics (28.2 % vs. 22.7 %), and fewer had hospital deliveries (49.4 % vs. 53.1 %). Similar patterns were also seen in women with <5^th^ percentile infants. These trends were similar to those noted in the pretrial period. Thus, although there were differences in care between the intervention and control clusters during the ACT trial, these differences preceded the trial. Therefore, we cannot rule out that differences in care were associated with the higher stillbirth rate in the intervention clusters.

**Table 4:** Factors related to process of care by ACT intervention group among all births

Characteristic	Pretrial Period		Trial Period	
	Intervention	Control	Intervention	Control
Deliveries, N	30,492	34,533	48,219	51,523
Antenatal Care				
Any antenatal care	28,743 (94.4)	32,801 (95.1)		
Number of antenatal visits	*Data not collected during pretrial period*		45,374	48,052
0			1,216 (2.7)	1,111 (2.3)
> 3			24,663 (54.4)	25,491 (53.0)
Trimester of 1st antenatal visit			43,980	46,632
1st			22,196 (50.5)	24,801 (53.2)
2nd			14,648 (33.3)	14,059 (30.1)
3rd			7,136 (16.2)	7,772 (16.7)
Administration of diagnosis tests or preventive care				
Syphilis or HIV test	21,944/30,435 (72.1)	24,071/34,446 (69.9)	37,975/47,961 (79.2)	40,343/51,185 (78.8)
Tetanus toxoid vaccine	26,892/30,422 (88.4)	30,467/34,478 (88.4)	40,313/47,980 (84.0)	44,453/51,219 (86.8)
Prenatal vitamin/iron	27,706/30,405 (91.1)	30,829/34,472 (89.4)	44,321/47,952 (92.4)	47,212/51,191 (92.2)
Delivery care				
Delivery attendant	30,490	34,531	48,215	51,519
Physician	10,305 (33.8)	12,709 (36.8)	19,122 (39.7)	23,233 (45.1)
Nurse/nurse midwife/LHW	10,348 (33.9)	10,094 (29.2)	18,166 (37.7)	15,366 (29.8)
TBA/Family/Unattended	9,837 (32.2)	11,728 (34.0)	10,927 (22.7)	12,920 (25.1)
Delivery location	30,482	34,494	48,217	51,519
Hospital	12,013 (39.4)	15,008 (43.5)	23,798 (49.4)	27,345 (53.1)
Clinic	8,486 (27.8)	7,619 (22.1)	13,593 (28.2)	11,675 (22.7)
Home/Other	9,983 (32.8)	11,867 (34.4)	10,826 (22.5)	12,499 (24.3)
C-section	3,001 (9.8)	3,279 (9.5)	7,133/48,218 (14.8)	7,655/51,520 (14.9)

We did not have data to evaluate whether fetal infection was more common in stillbirths in the control or intervention clusters since autopsies or placental examinations were not performed. However, as in the primary paper, we had data to evaluate potential infection in the mothers. Suspected maternal infection was reported in 2.5 % of women in the intervention clusters and 1.7 % in the control clusters (OR 1 · 45, 95 % CI 1 · 33–1 · 58, *p* < 0 · 0001). Among women who delivered <5^th^ percentile infants, the suspected maternal infection was reported in 10.0 % of the women in the intervention clusters and 6.4 % of women in the control clusters (OR 1 · 67, 95 % CI 1 · 33–2 · 09, *p* < 0 · 0001). Thus, there appeared to be a greater incidence of maternal infection in the intervention clusters.

Mothers of stillbirths were also more likely to be classified as having a suspected maternal infection (207/2,526 (8.2 %) versus 1,867/97,211 (1.9 %) of mothers with a live birth. Among mothers with stillbirth, 114/1,280 (8.9 %) in the treatment group and 93/1,153 (7.5 %) in the control group experienced suspected maternal infection. Three of the sites (Argentina [12.0 % vs 6.7 %], Belgaum [7.8 % versus 5.3 %] and Nagpur [11.7 % vs 5.0 %] showed modest higher risk of having a suspected maternal infection in the treatment arm. The other four sites had less than a 1.2 % difference in risk between the arms withGuatemala and Kenya having slightly higher risk in the treatment arm and Zambia and Pakistan having slightly higher risk in the control arm. Because the number of events was relatively small and all sites had heterogeneity of effects across randomization strata, no formal inference of overall or within site differences was conducted and an overall odds ratio was not calculated.

Finally, we tried to determine if the increase in stillbirth could be related to the receipt of ACS. In the intervention clusters, the mothers of 293/1,267 stillbirths (23.2 %) received ACS while in the control clusters, the mothers of 23/1,237 stillbirths (1.9 %) received ACS (Table 5). In the intervention clusters, fetuses whose mothers received ACS had more than twice the stillbirth rate compared to those who did not (51.1 vs 24.3 per 1,000 births). In the control clusters, the stillbirth rate for babies whose mothers received ACS was more similar to those who did not (29.2 vs 25.4 per 1,000 births). Thus, while maternal receipt of ACS in the intervention clusters was associated with a doubling of the stillbirth rate, in the control clusters the increase in stillbirth rate among those fewer women who received ACS, while still in the same direction, was not as great as in the intervention clusters. Nevertheless, with the mothers of 23.5 % of stillbirths receiving ACS, more than enough women received ACS to potentially account for the higher stillbirth rate in the intervention clusters.

**Table 5:** Stillbirth rates by ACS administration^a^ and ACT intervention group and site

	Argentina				Guatemala				Pakistan				Belgaum			
	Treatment		Control		Treatment		Control		Treatment		Control		Treatment		Control	
Characteristic	ACS	No ACS	ACS	No ACS	ACS	No ACS	ACS	No ACS	ACS	No ACS	ACS	No ACS	ACS	No ACS	ACS	No ACS
Deliveries, N	271	1,297	163	1,551	540	5,135	39	3,812	1,871	5,927	125	8,186	1,743	13,228	332	16,407
Stillbirths, N	5	18	3	24	12	94	0	69	117	271	4	347	80	296	4	377
Stillbirths ≥ 20 weeks GA or ≥ 500 g, N (Rate/1000)	5 (18.5)	18 (13.9)	3 (18.4)	24 (15.5)	12 (22.2)	94 (18.3)	0 (0.0)	69 (18.1)	117 (62.5)	271 (45.7)	4 (32.0)	347 (42.4)	80 (45.9)	296 (22.4)	4 (12.0)	377 (23.0)
	Nagpur				Kenya				Zambia				Total			
	Treatment		Control		Treatment		Control		Treatment		Control		Treatment		Control	
Characteristic	ACS	No ACS	ACS	No ACS	ACS	No ACS	ACS	No ACS	ACS	No ACS	ACS	No ACS	ACS	No ACS	ACS	No ACS
Deliveries, N	612	5,822	84	6,351	178	5,708	30	7,007	519	3,043	15	4,550	5,734	40,160	788	47,864
Stillbirths, N	36	110	5	164	16	123	5	148	27	62	2	85	293	974	23	1,214
Stillbirths ≥ 20 weeks GA or ≥ 500 g, N (Rate/1000)	36 (58.8)	110 (18.9)	5 (59.5)	164 (25.8)	16 (89.9)	123 (21.5)	5 (166.7)	148 (21.1)	27 (52.0)	62 (20.4)	2 (133.3)	85 (18.7)	293 (51.1)	974 (24.3)	23 (29.2)	1,214 (25.4)

^a^Data on administration of ACS from the MNH Registry are available for 1,267/1,304 (97.2 %) of stillbirths in the intervention group and 1,237/1,264 (97.9 %) of stillbirths in the control group

## Discussion

We explored potential reasons for the higher stillbirth rate in the intervention clusters compared to the control clusters in the ACT trial. As a secondary analysis of a non-primary outcome in a trial not designed for this purpose, the results cannot be considered definitive, but may suggest further research directions.

The higher stillbirth rate in intervention clusters was found in five of the seven sites. Although the overall differences in stillbirth rates between the intervention and control clusters were significant, the results were not in the same direction at all sites. Further exploration of the reasons for site-specific differences in stillbirth rates therefore may be warranted. Stillbirth rates were higher in the intervention clusters to the same degree in male and female fetuses so differential impact by gender does not appear to be part of the explanation for these findings. The increase in stillbirths occurred in fetuses labeled as macerated and not in fresh stillbirths. This finding suggests that the higher stillbirth rate in the intervention clusters did not occur during the labor and delivery process, but instead occurred in the antenatal period. Since about 50 % of the mothers of stillbirths delivered within a week after receiving the ACS injection, this suggests, but does not prove, that some of the increase in stillbirths associated with being in the intervention clusters occurred within days after receiving the ACS injection, but not during labor. On the other hand, about 50 % of the mothers of stillbirths delivered more than a week following receipt of ACS.

Because of the relatively poor gestational age ascertainment at the sites during the trial, the results regarding gestational age and stillbirth rates should be interpreted cautiously. Nevertheless, the stillbirth rate was higher in the intervention clusters in fetuses who appeared to be term compared to preterm, in fetuses ≥25^th^ percentile birth weight, and in fetuses ≥ 2500 g. These consistent results strongly suggest that there were higher stillbirth rates in the heavier more mature fetuses in the intervention clusters. The results for the smaller and earlier gestational age fetuses are less consistent. Since there were fewer stillbirths in the intervention clusters in the < 5^th^ percentile births, in the < 1000 g births and in the 1500 to 2499 g births and in fetuses categorized as preterm, it is unlikely the intervention was associated with a higher stillbirth rate in smaller earlier gestational age stillbirths, and there may have been a small reduction in the stillbirth rates in these groups.

The reason mothers of fetuses who received ACS delivered outside the targeted window or even at term are unknown. One possible explanation is that the gestational age dating was often inaccurate, and the care givers who determined use of ACS were often inexperienced and poorly trained. Another explanation, and a common finding in many of the published ACS trials, is that a significant proportion of ACS recipients were in the appropriate gestational age interval when they received the ACS injection, but delivered later in their pregnancy [11, 12]. We expect that both explanations account for the fact that many women in this trial who received ACS delivered heavier and older gestational age infants.

We have little data to help us explain the cause for the higher stillbirth rate in the intervention clusters. From the primary paper we know that women in the intervention arm likely had more infections than women and neonates in the control clusters [2]. We also know that the increase in stillbirths in the intervention compared to control clusters was associated with a higher rate of maceration, suggesting that asphyxia during labor was not the cause of the higher stillbirth rate. Since aside from asphyxia and congenital anomalies, infection is the primary cause of stillbirths, it is conceivable that the higher rate of stillbirths associated with being in the intervention clusters is in part related to increased infection. Proving that this is the explanation for the higher rate of stillbirths in the intervention clusters is not possible from the available data, but any future study of ACS in LIC settings should collect data related to maternal and fetal infection status.

There were differences in obstetric care between the intervention and control clusters and these differences were found in both the pretrial and trial period. In the pretrial period these differences were also associated with a difference in stillbirth rates between the intervention and control clusters. Therefore, our data do not allow us to rule out the possibility that part of the differences in stillbirth rates between the intervention and control clusters prior to and during the trial were due to differences in obstetric care.

Most randomized trials of ACS have not reported stillbirth rates and those that have reported stillbirth rates generally report stillbirths that occurred in the week following ACS administration or in preterm newborns. However, a meta-analysis of women who received ACS and delivered later than 7 days after ACS administration showed significant increases in both perinatal deaths and in chorioamnionitis in the treatment group [13]. Stillbirths were increased, but not significantly so. Thus, there is a precedent in the literature that ACS given to women who ultimately deliver outside the one-week window of likely benefit for neonatal outcomes may have an increase in infection and stillbirth associated with ACS administration. Because so many women in this trial delivered outside the 7-day window, and delivered more mature and heavier infants, these factors may explain some of the ACT study stillbirth findings. However, more likely, the differences in stillbirth rates between the intervention and control clusters in the ACT trial period are explained at least in part by the differences in health care that existed prior to the ACT study and continued throughout the study.

## Conclusions

In summary, in the ACT trial there was a small but significantly higher rate of stillbirths among women in the intervention clusters. The increase was seen in 5 of the 7 sites, and was not consistent across the study. Differences in care between the intervention and control clusters may explain part or all of the higher stillbirth rate in the intervention clusters, but this possibility cannot be proven from the available data. It is also possible that differences in unmeasured maternal characteristics such as syphilis or malaria played a role. The higher stillbirth rate in the intervention clusters was similar in male and female fetuses. The higher stillbirth rate in the intervention clusters was apparent only in the larger and term fetuses. There was no consistent increase in stillbirths in the intervention clusters in the lighter more preterm infants and there may have been a small reduction in stillbirths in some birth weight and gestational age groups. Because of the poor gestational age dating available for those participating in the ACT trial, we can make no definitive statement about the impact of the intervention on stillbirth rates in smaller and earlier gestational age fetuses. Finally, data related to causation are limited, but suggest that fetal asphyxia is not likely to explain our findings. Instead, our data suggest that infection may explain some of the observed higher rate of stillbirths in the intervention clusters. The meta-analysis of individually randomized ACS trials showing an increase in infection and perinatal mortality in women delivering more than 7 days after receiving ACS lends credence to this hypothesis [10]. Nevertheless, it appears that the higher stillbirth rate in the intervention clusters preceded the study and was coincident with differences in obstetric care between the intervention and control clusters. We also emphasize that the difference in stillbirth rates between the intervention and control clusters was small and although statistically significant could have occurred by chance.
